# Comparison of ARIMA, ES, GRNN and ARIMA–GRNN hybrid models to forecast the second wave of COVID-19 in India and the United States

**DOI:** 10.1017/S0950268821002375

**Published:** 2021-11-02

**Authors:** Gang Wang, Tiantian Wu, Wudi Wei, Junjun Jiang, Sanqi An, Bingyu Liang, Li Ye, Hao Liang

**Affiliations:** 1 Guangxi Key Laboratory of AIDS Prevention and Treatment, School of Public Health, Guangxi Medical University, Nanning, Guangxi, China; 2 Department of Periodontics and Oral Medicine, College of Stomatology, Guangxi Medical University, Nanning 530021, Guangxi, China; 3 Life Sciences Institute, Guangxi Medical University, Nanning, Guangxi, China

**Keywords:** ARIMA, ARIMA–GRNN, COVID-19, ES, GRNN

## Abstract

As acute infectious pneumonia, the coronavirus disease-2019 (COVID-19) has created unique challenges for each nation and region. Both India and the United States (US) have experienced a second outbreak, resulting in a severe disease burden. The study aimed to develop optimal models to predict the daily new cases, in order to help to develop public health strategies. The autoregressive integrated moving average (ARIMA) models, generalised regression neural network (GRNN) models, ARIMA–GRNN hybrid model and exponential smoothing (ES) model were used to fit the daily new cases. The performances were evaluated by minimum mean absolute per cent error (MAPE). The predictive value with ARIMA (3, 1, 3) (1, 1, 1)_14_ model was closest to the actual value in India, while the ARIMA–GRNN presented a better performance in the US. According to the models, the number of daily new COVID-19 cases in India continued to decrease after 27 May 2021. In conclusion, the ARIMA model presented to be the best-fit model in forecasting daily COVID-19 new cases in India, and the ARIMA–GRNN hybrid model had the best prediction performance in the US. The appropriate model should be selected for different regions in predicting daily new cases. The results can shed light on understanding the trends of the outbreak and giving ideas of the epidemiological stage of these regions.

## Introduction

As acute infectious pneumonia, the new coronavirus disease-2019 (COVID-19) has created unique challenges for each nation and region. According to the latest World Health Organization (WHO) figure, as of 30 May 2021, there were a total of 169 597 415 confirmed COVID-19 cases worldwide, with a death toll of 3 530 582. With regard to US, there have been 32 916 501 confirmed cases, including 588 292 deaths, ranking the first among all countries (https://covid19.who.int/table). In the US, the first confirmed case was detected on 20 January 2020; On 23 March 2020, the number of new cases exceeded 15 000 for the first time, and the number of new cases reached a peak of 312 247 on 10 January 2021 [1]. In India, the second wave of COVID-19 become more serious. Since 1 April 2021, the number of infections and deaths has increased exponentially and become one of the most important concerns of India and all over the world.

The control of the COVID-19 epidemic in India affects the global prevention and control effect. Therefore, it is of great significance to propose and fit a model to forecast the epidemic trend of the COVID-19 based on real-time monitoring data [2, 3]. Evaluating the impact of COVID-19 on society is important and urgent [4]. Epidemiological time series forecasting plays an important role in disease surveillance, because it allows the managers to develop strategic planning, which helps to avoid a large scale of the epidemic [5]. At present, many mathematical models, including regression analysis method, time series analysis method, and neural network technology, have been applied to predict the incidence of infectious diseases [6–9]. The autoregressive integrated moving average (ARIMA) model is a time series analysis method firstly proposed by Box and Jenkins in the 1970s, which works based on linear theory; the model mainly captures a linear relationship and assumes the normality of errors [10]. However, it is not appropriate to study non-linear or unstable phenomena [11, 12]. The generalised regression neural network (GRNN) model, which has a unique ability of accelerated learning and greater capability for non-linear fitting, has been widely used in disease prediction, modelling and estimating, especially when the data series is unstable [13]. The exponential smoothing (ES) model is also a kind of method which takes the historical information into comprehensive consideration [14, 15].

In this study, we aimed to fit an ARIMA model, GRNN model, ES model and ARIMA–GRNN hybrid model to fit and predict the daily new cases of COVID-19 in India and the United States. The performance of the models was compared. As a result of comparative analysis, the best-fit model was determined. Prospective forecasting studies were carried out for daily new COVID-19 cases based on the best-fit models. By comparing the epidemic trend in both India and the United States, we aimed to find the pattern and relationship between India and the United States with regard to the second wave of COVID-19.

## Methods

### Ethical approval

The data were collected from public databases. Formal ethical approval was not required for this study.

### Data source

In this study, the raw data of daily new cases of COVID-19 from 1 March 2020 to 27 May 2021 in India and the United States were obtained from the public-access databases of WHO (https://covid19.who.int) and Our World in Data (https://ourworldindata.org/). The data from 1 March 2020 to 13 May 2021 of newly confirmed cases were used to train the models and the data from 14 May to 27 May were used to validate the models.

### Construction of ARIMA model

The ARIMA model was fitted using JMP 14 Pro and Stata 16 software. The construction of an ARIMA model included four steps, as follows: Initially, the variables specified in the model for estimation were tested for stationarity. Secondly, the autocorrelation function (ACF) analysis and the partial autocorrelation function (PACF) analyses were used to determine the possible values of *p*, *q*, *P*, and *Q*. Thirdly, in the diagnostic checking step, parameter estimation was carried out to determine whether they were statistically significant [16]. The Akaike information criterion (AIC) and Schwarz Bayesian information criterion (SBC) were used to determine the optimal model. In addition, The Ljung−Box Q test for the residual series of the model was conducted to determine if the residual series was a white-noise sequence (*P* > 0.05). Finally, the data were forecasted according to the selected ARIMA (*p*, *d*, *q*) (*P*, *D*, *Q*) *s* model. In this study, considering the periodicity of time series, the seasonal ARIMA (SARIMA) model was constructed, which is an extension of the ARIMA model.

### Construction of GRNN model

The GRNN model was fitted using Matlab software as previously described [13]. The GRNN model proposed by Specht was a mathematical model based on nonlinear regression analysis [17]. The structure of the GRNN model consisted of four layers: input layer, mode layer, summation layer and output layer [13, 18–20]. For each input vector *X*, there was an output vector *Y* corresponding to it. The formula of the analysis was as follows:
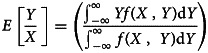


Among them, *X* represented the input vector, and *Y* represented the predicted value of GRNN. *E*[*Y/X*] represented the expected output vector Y corresponding to an input vector X, and *f* (X, Y) represented the joint probability density function of X and Y. The structure of the GRNN model could be expressed as (*N*−1) GRNN model, which was a GRNN model with *N*-dimensional input and 1-dimensional output. In addition, an optimised GRNN model was constructed by adjusting the smoothing parameters. Therefore, the N-value and the smoothing parameters were important for determining the quality of the model. To establish the optimal GRNN model, the optimal parameter values needed to be found. First of all, the original data were divided into two parts: the training set, and the validation set. Secondly, a series of GRNN models were constructed by using different N-values and smoothing factors. The optimal GRNN model was selected with the minimum mean absolute per cent error (MAPE) value. Finally, the number of new cases was forecasted according to the selected GRNN model.

### Construction of ES model

We explored the ES model using JMP 14 Pro software as previously described [21]. The ES model proposed by Robert G. Brown was a technique for manipulating data from a series of chronological observations to downplay the effects of random variation [22–24]. The basic formula of the analysis was as follows:
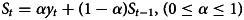


The Ljung−Box Q-test was calculated to test the hypothesis concerning whether the residual sequence was an independent sequence [25]. Time-series models could be used if the residual sequence was a white noise sequence [26]. The statistic stationary *R^2^*, represented the stable coefficient of determination, was used to compare the difference between the fixed components and the simple mean model. When the value was positive, negative, or zero, it meant the model was superior, inferior, or equivalent to the simple mean model [27]. At the same time, the optimal model was further established according to the minimum principle of MAPE.

### Construction of ARIMA–GRNN model

The ARIMA–GRNN hybrid model was built using Matlab software as described previously [13]. We used the fitting data of the ARIMA model as the input variable and the actual data as the manipulated value to fit the hybrid ARIMA–GRNN model. The smoothing factor *σ* was the only regulation parameter. Specht proposed a method to find the optimal smoothing factor, that was, randomly selected two samples (each sample may be selected and the probability is the same) as the test data, and the other samples were used to train the network. By testing a series of smoothing factors of the training network, the best smoothing factor with the minimum *MAPE* of the network was selected [28]. Finally, the predicted values created by the ARIMA model were used as the input data of the hybrid model, so then the combined model could output the predictive values [29]. It is worth mentioning that we developed the graphical user interface (GUI) of the ARIMA–GRNN hybrid model based on the Matlab platform, which have been applied in practice and have been recognised and supported by many researchers. (https://ww2.mathworks.cn/matlabcentral/fileexchange/61383-arima-grnn-hybrid-model?s_tid=srchtitle_ARIMA-GRNN_1).

## Results

### Characteristics of daily new cases

**Fig. 1.**
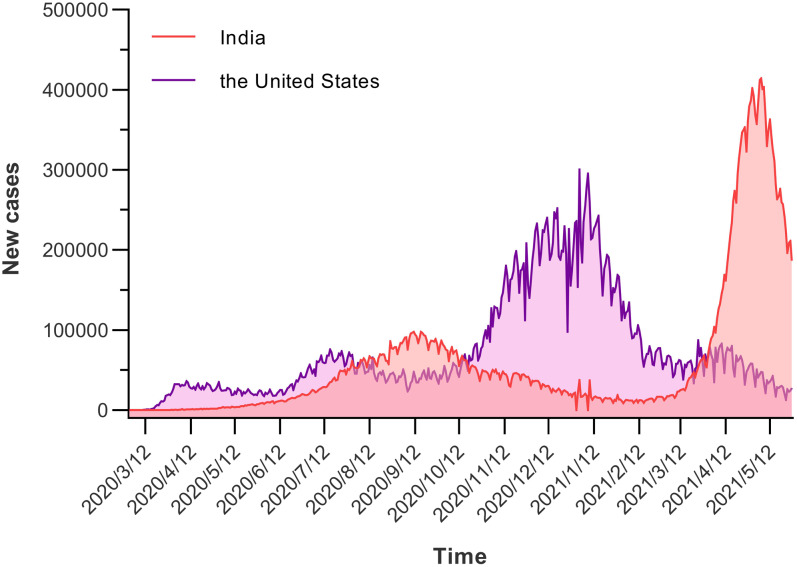
Daily new cases of COVID-19 in the United States and India (from 1 March to 7 November 2020). The number decreased gradually from 1 April 2020 until 11 June 2020 (from 32 259 to 23 133), the epidemic begins to rise gradually on 12 June 2020 with the first peak number of cases on 19 July 2020 and a decrease in the number of cases till 10 September 2020. In India, the number of new cases exceeded 100 000 per day on 4 April 2021 for the first time. On 6 May 2021, the number exceeded 410 000, reaching the peak of the second wave, and then began to decrease.

The United States was the country with the highest number of cumulative cases and daily new cases so far. With regard to the new cases emerged every day, the number decreased gradually from 1 April 2020 until 11 June 2020 (from 32 259 to 23 133), the epidemic began to rise gradually on 12 June 2020 with the first peak number of cases on 19 July 2020 and decrease in the number of cases till 10 September 2020. The number of new cases continued to increase after 10 September 2020, exceeding the first peak. The development of the epidemic peaked until 2 January 2021, and the number of new cases per day reached 300 310. The epidemic trend in India was highly comparable with that in the US, while the occurrence time of epidemic peak was different. On 4 April 2021, the number of new cases exceeded 100 000 per day for the first time. On 6 May 2021, the number of new cases per day exceeded 410 000, reaching the peak of the second wave, and then began to decrease. The trends in the prevalence of COVID-19 in India and the United States were shown in Figure 1.

### Construction of ARIMA and evaluating the performance of the models

**Fig. 2.**
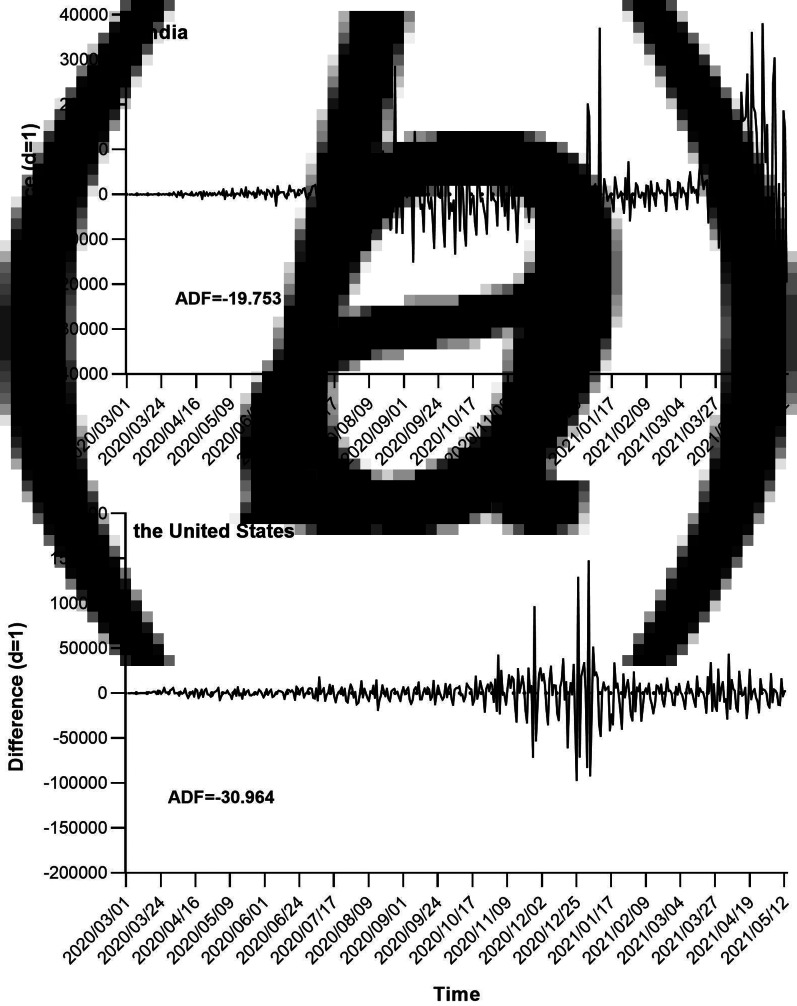
Time series after one difference (*d* = 1). After data processing, the origin time series become stationary, that is the ADF test showed a statistically significant result.

For constructing the ARIMA model, daily COVID-19 new cases from 1 March 2020 to 13 May 2021 were used as the training set; Data from 14 May 2021 to 27 May 2021 was used to validate the prediction ability of these models. Figure 1 showed the daily new cases of COVID-19 in India and the United States; briefly, the original sequences showed a downward or upward trend with a seasonal cycle rhythm, which were not smooth, indicating that the augmented Dickey–Fuller (ADF) test was no statistical difference. Therefore, a transformation with a difference (*d* = 1) was processed to the origin time series to remove numerical instabilities. The sequences after the difference were shown in Figure 2. After data processing, the ADF test showed a statistically significant result (*P* < 0.001), indicating that the time series was stationary (Supplementary Table S1).

**Fig. 3.**
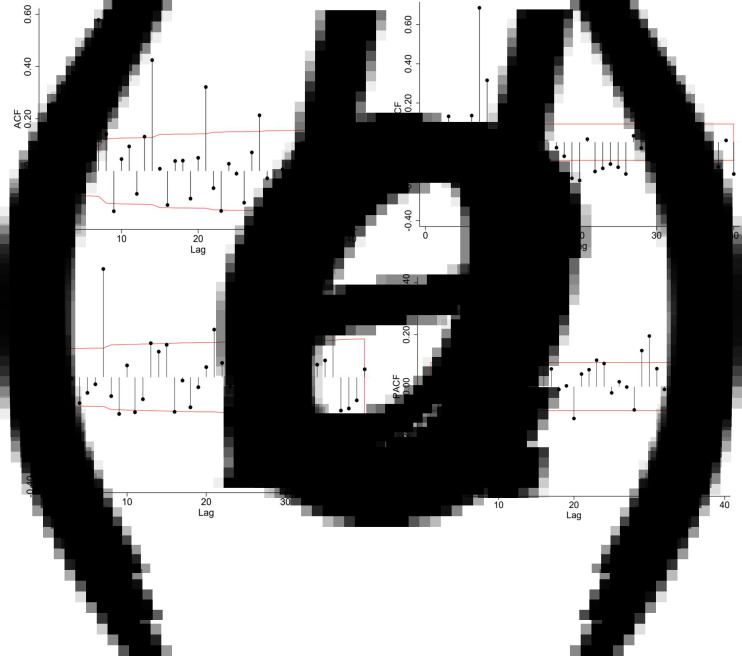
ACF and PACF of transformed COVID-19 new cases series. ACF: the autocorrelation function graph; PACF: the partial autocorrelation function graph.

Then, the ACF and PACF analyses were developed to determine the parameters of the ARIMA models (Fig. 3). In India, three appropriate models, namely, ARIMA (3, 1, 3)(3, 1, 3)_14_, ARIMA (3, 1, 3)(1, 1, 1)_14_, and ARIMA (3, 1, 3)(3, 1, 1)_14_, were identified to effectively predict the COVID-19 new cases according to the results of ACF and PACF. These three models had approximated AIC and Schwarz Bayesian criterion (*SBC*) values (Supplementary Table S2); however, the model ARIMA (3, 1, 3)(3, 1, 3)_14_ had lower *AIC* value than the other two while model ARIMA (3, 1, 3)(1, 1, 1)_14_ had lower *SBC* and mean absolute percentage error (MAPE) values. Similarly, three appropriate models, namely, ARIMA (2, 1, 3)(0, 1, 1)_14_, ARIMA (2, 1, 3)(2, 1, 1)_14_, and ARIMA (3, 1, 3)(3, 1, 2)_14_, were identified to effectively predict the COVID-19 new cases, and the results showed that ARIMA (2, 1, 3)(0, 1, 1)_14_ had the lowest *AIC* and ARIMA (2, 1, 3)(2, 1, 1)_14_ demonstrated the optimal predictive performance. The time series after one difference (*d* = 1) in India and the United States were shown in Figure 2, the *AIC, SBC*, and *R*^2^ of the three appropriate ARIMA models were presented in Supplementary Table S2, and the residual scatter and ACF plots were shown in the Supplementary Figure S1.

### Construction of GRNN and ES and evaluating the performance of the models

**Fig. 4.**
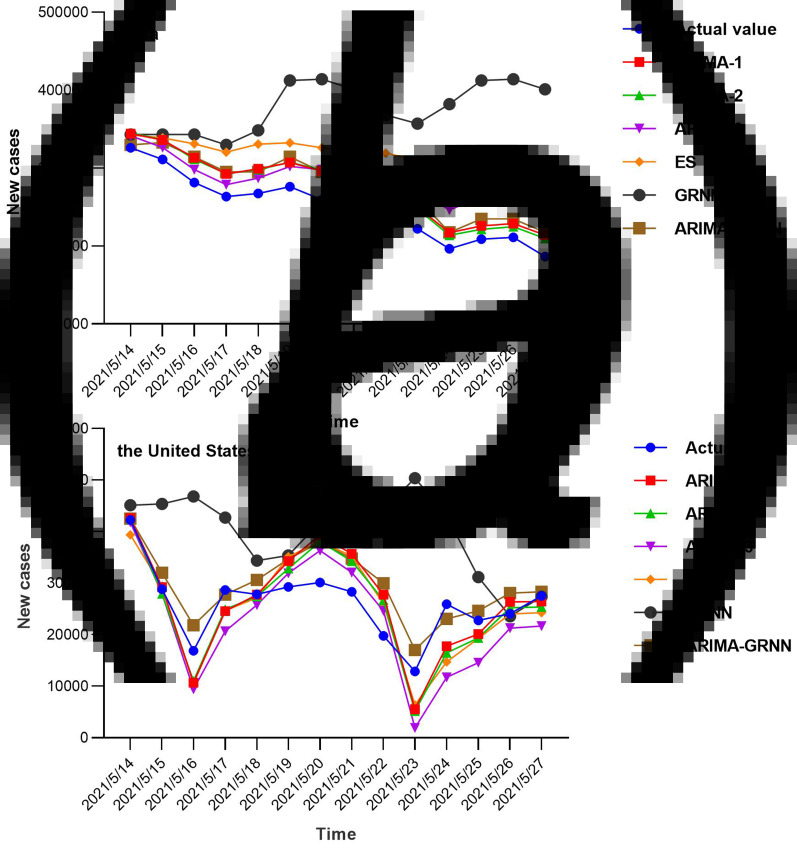
Forecasting curves of the different models as well as the actual COVID-19 new cases in India and the US. In India, ARIMA1 refers to ARIMA (3, 1, 3) (3, 1, 3) _14_, ARIMA2 refers to ARIMA (3, 1, 3) (1, 1, 1) _14_, and ARIMA3 refers to ARIMA (3, 1, 3) (3, 1, 1) _14_; ES refers to exponential smoothing. Compared with other models, the forecasting value of ARIMA2 was closer to the actual value. In the US, ARIMA1 refers to ARIMA (2, 1, 3) (0, 1, 1) _14_, ARIMA2 refers to ARIMA (2, 1, 3) (2, 1, 1) _14_, and ARIMA3 refers to ARIMA (3, 1, 3) (3, 1, 2) _14_. The ARIMA–GRNN hybrid model was closer to the actual value.

The number of daily COVID-19 new cases was forecasted using the GRNN model and ES model, respectively. The GRNN model, a type of radial basis function neural network, was constructed with Matlab programming, the time series from 1 March 2020 to 13 May 2021 were selected to develop the network, the optimal *N* values of GRNN were four and 12 in India and the US, respectively, indicating basic model with four-dimensional (12-dimensional in the US) input and one-dimensional output had the minimum MAPE compared to other *N* values. The forecasting curve of the optimal GRNN model and actual COVID-19 new cases curve is shown in Figure 4 and the performance metrics (*MAPE*) is presented in Table 1.

**Table 1.** Predictive performance of different models

Region	ARIMA-1	ARIMA-2	ARIMA-3	ES	GRNN	ARIMA–GRNN
India	0.109	**0.102**	0.169	0.307	0.522	0.117
the US	0.200	0.193	0.257	0.204	0.672	**0.173**

Bold value is the minimum MAPE value, which indicates that the model has the optimal predictive performance.

With regard to the time series in India, the horizontal smoothing weight, trend smoothing weight and seasonal smoothing weight were 0.829, 0.128 and 0.761, respectively (Supplementary Table S3). Compared with the ARIMA model, the AIC and SBC of the ES method were higher. In the US, the performance of the ES model was also inferior to the ARIMA model, of which the horizontal smoothing weight, trend smoothing weight and seasonal smoothing weight were 0.353, 0.060 and 0.138, respectively.

### Construction of hybrid ARIMA–GRNN and performance evaluation

The fitted new cases values of COVID-19 from the optimal ARIMA model were selected as inputs of the GRNN model, and the actual values were used for subsequent testing of the final model. To determine the best smoothing factor, the number of daily new cases from 1 March 2020 and May 2021 were randomly used as the testing samples for the GRNN model. The results showed that 0.008 was the optimal smoothing factor in the time series of India, and 0.011 in the US. The ARIMA–GRNN model showed lower MAPE values when predicting the number of new cases in the United States (Table 1).

### Forecasting future daily new COVID-19 cases using six models

**Fig. 5.**
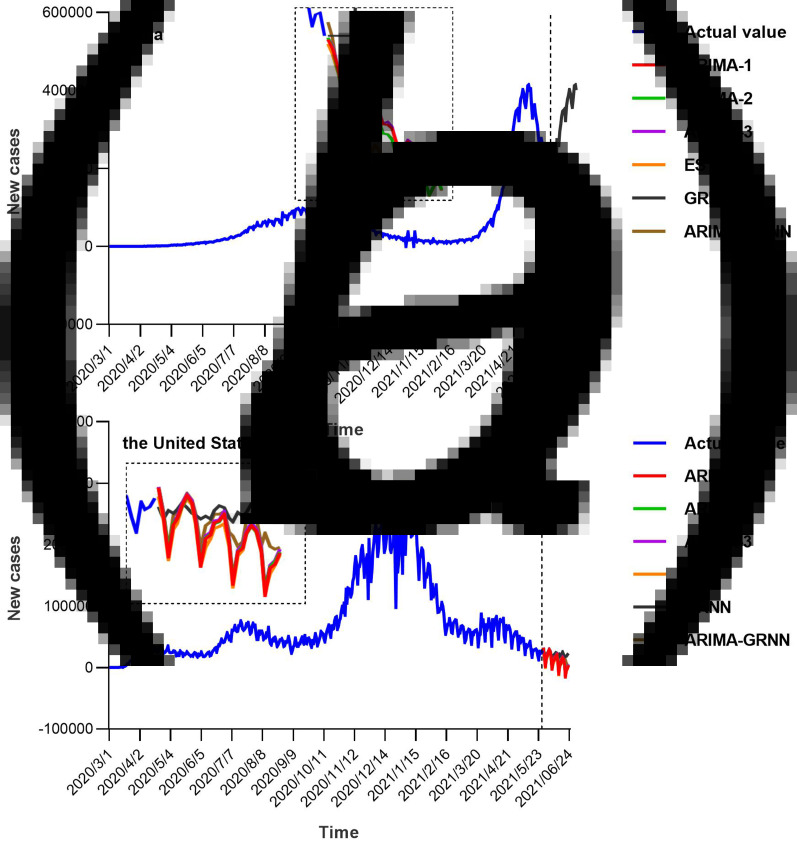
Forecasting curves of the future new COVID-19 cases by six models. After 27 May 2021, the epidemic in both countries showed a decreasing trend and the situation would be moderated. Two small areas were enlarged to show the details of the forecasting curves (black dashed box).

The models were identified by AIC and SBC, and the number of future COVID-19 daily new cases in India and the United States were predicted according to these models. The fitting and predictive curves were shown in Figure 4. In all fitting curves, the predictive value with ARIMA (3, 1, 3) (1, 1, 1) _14_ model was closer to the actual value compared to the other four models in India, while the ARIMA–GRNN presented a better performance in the time series of the US. Figure 5 showed the prediction curves to capture the future trend of COVID-19 new cases in India and the United States using the different models. After 27 May 2021, the epidemic in both countries showed a decreasing trend and the situation would be moderated.

## Discussion

Autumn and winter are the seasons for a high incidence of respiratory infectious diseases, and the risk of a second outbreak of the COVID-19 epidemic cannot be ignored. According to the report, the second wave of the COVID-19 pandemic in India has arrived, and the impacts seem to be more severe. Therefore, it is of great significance to establish an effective monitoring network that can help governments and other stakeholders to control the further spread of the COVID-19 epidemic [30]. In the study, GRNN, ARIMA, ES and ARIMA–GRNN models were established using the historical COVID-19 data, and their predictive abilities were compared. The ARIMA model may have a better performance in terms of metrics in predicting the new cases of COVID-19 epidemic in India, while the ARIMA–GRNN model in the US. The ARIMA model, a well-known time series prediction method which emphasises on analysing stochastic and probabilistic properties of time-series data, is widely used to predict the initiation and epidemic progression of infectious diseases. According to the results, the ARIMA model showed its advantage in predicting new cases in India. With regard to the GRNN model, it was developed as a new potential tool for the prediction of infectious diseases. It is characterised by a fast convergence and good non-linear approximation performance based on a radial basis network. However, the computational and spatial complexity could not be ignored since each test set needs to be calculated, and all train sets need to be stored. In our study, the GRNN model obtained the biggest AIC and SBC values, and exhibited a poor performance both in India or the US, indicating that traditional machine learning methods may be more effective in terms of predicting time series compared to deep learning-based neural networks. Therefore, we speculated that the relatively poor performance of GRNN was firstly due to the time series structure itself and also due to the existence of the overfitting problem. However, in the US, the ARIMA–GRNN model had the lowest MAPE value, indicating the selection of the optimal model and monitoring methods were different when confronted with different monitoring data.

Many researchers have used different models to predict the epidemic of COVID-19. A study used the logistic growth curve model to evaluate the change level of the number of confirmed cases before and after the implementation of emergency response. Moreover, historical data were extracted to simulate short-term dynamic prediction, and explore the application of the logistic growth curve model in the assessment of COVID-19. The results showed that the model had good prediction accuracy, but there were differences in predicting the number of confirmed cases in different provinces, indicating that the ability of the models to deal with different epidemic curves was inconsistent [31]. Another study constructed a simplified discrete-time model to predict the number of covid-19 confirmed cases in different countries in the next 14 days based on the discrete-time equation. The model was simple and had only two parameters (ɑ and *β*), which facilitated its implementation by health sector personnel and interested people in general. Nevertheless, instead of extensively covering the possible complexities associated with disease transmission, the model focused on the main processes of transmission [32]. In the early stage of the disease epidemic, some researchers used the ARIMA model to estimate the prevalence of covid-19 in Italy, Spain and France [33]. However, the study showed that the minimum MAPE values of Italy, Spain and France were 4.752, 5.849 and 5.849 respectively, which were greater than the MAPE values of the model constructed in this study. Besides, multivariate linear regression [34], grey forecasting models [35], backpropagation neural networks [16], simulation models [36] and SEIR [37] were also commonly used to predict the prevalence of COVID-19. Remarkably, epidemics are affected by many different factors, and the general spread of the outbreak is characterised by tendencies and randomness, which makes them difficult to be widely understood and implemented. Therefore, the mentioned statistical tools are not enough to analyse the randomness of epidemics, and the models are difficult to generalise. The selection of predictive models should exchange complexity for simplicity in its comprehension and implementation while maintaining satisfactory predictive capability, which is also the core principles for its assimilation by the public health sector.

India and the United States epidemic have experienced the outbreak of the second wave. We think there might be the same trend between the two regions, which may be one of the ways to predict the epidemic. First of all, the second outbreak episode in the United States was due to the crowd gathering activity and unavailability of adequate personal protective measures. Similarly, the sudden epidemic widens in India was due to the same reason. In addition, the epidemic curve in the two regions (Fig. 1) showed that the peak of the second epidemic in India was 123 days later than that in the US. However, the epidemic curves of the two regions were basically consistent when the two peaks were coincided. This phenomenon showed that countries with the late emergence of the second wave of COVID-19 could reference the epidemic curve and intervention policies in areas where the epidemic occurred earlier to formulate prevention measures and treatment methods suitable for the region. Therefore, we believe that the epidemic trend in different regions can be compared to comprehensively analyse the epidemic situation in the monitoring and prediction of the epidemic, and relevant measures can also be formulated to control the outbreak of the epidemic.

The daily new case of India seemed to decrease according to the predictive result, it seemed to be good news for prevention and control in India. However, some international media believed that due to the long-standing phenomenon of insufficient inspections in some parts of India, the number of newly confirmed cases and death cases might be underestimated. Some experts analysed and reported that the actual number of newly confirmed cases and death cases in India might be several times higher than the official data. Therefore, efforts are still needed to prevent and control the COVID-19 epidemic in India. A sustained increase in the epidemic has a major impact on the economy of India, COVID-19 exacerbates inequalities and also exposes inequities in a health system. The first is that there are still loopholes in vaccination. Only 3% of India's population has been fully vaccinated, ranking the lowest among the 10 countries with the most cases. As India's production activity recovers and many rural people return to cities, a third round of outbreaks may occur. The second hidden danger is that the ‘double mutant virus’ (B.1.617 and B.1.618 variants) found in India has spread all over the world. WHO showed that more than 90 countries and regions have found a ‘double mutant virus’. Therefore, for epidemic prevention and control, the most important thing is to expand vaccination, which is also the only way to continuously reduce the spread of COVID-19, and the vaccination must be accelerated worldwide. The virus continues among unprotected populations as long as vaccine coverage has serious inequality.

With regard to the prediction and monitoring of COVID-19 in India and the US, there are still some potential differences between the predicted values and actual values due to complicated factors, including environmental factors, behavioural factors, lifestyle habits, cultural backgrounds, political factors and the intensity of intervention in different areas, etc. In the ARIMA model and ARIMA–GRNN model, the time series is the only variable. Additionally, when building the ARIMA model, the residual error did not pass the Ljung−Box test, although this was often found in practice. We believe that the Ljung−Box test statistic is constructed under the assumption that the sequence meets the homogeneity of variance. When the heteroscedasticity is detected in the sequence, the Ljung−Box statistic does not approximately obey the *Chi-square* distribution. Therefore, when conditional heteroscedasticity occurs, the Ljung−Box test result is no longer accurate, which shows that the correlation coefficient of the residual sequence is very small, and the sequence can be approximated as white noise, although the *P* value of the Ljung-Box test results is also small. Alternatively, we constructed the ARIMA model using different parameters, and their residual tests also showed non-white noise sequences, indicating that ARIMA's information extraction of this time series had reached its limit. Overall, although the ARIMA model has relatively good effectiveness and predictive ability in the prediction of COVID-19 daily new cases in India, and the ARIMA–GRNN model in the US, more advanced infectious disease forecast models, including multiple influence factors, are needed to make the forecast more precise and closer to the actual values.

## Conclusion

In our study, the prediction models, namely ARIMA, GRNN, ARIMA–GRNN model and ES were established and compared for their predictive abilities. ARIMA model was a better predictive model than others in forecasting daily COVID-19 new cases in India, and the ARIMA–GRNN hybrid model has better prediction performance in the US. In the monitoring and prediction of the epidemic, the epidemic trend in regions can be compared to comprehensively analyse the epidemic situation, and relevant measures can also be formulated according to the causes of the epidemic in other regions to control the outbreak of the epidemic. In addition, the performance of varying models in predicting daily new cases in disparate countries is different, appropriate models should be selected based on the incidence data of different regions.

## Data Availability

The data used or analysed during the current study are available from the corresponding author on reasonable request.

## Supplementary material

For supplementary material accompanying this paper visit http://dx.doi.org/10.1017/S0950268821002375. click here to view supplementary material
