# Supplementary material for: Comparison of ARIMA, ES, GRNN and ARIMA–GRNN hybrid models to forecast the second wave of COVID-19 in India and the United States
Source: Epidemiol Infect. 2021 Nov 2;149:e240. doi: 10.1017/S0950268821002375 (PMC8632421; doi:10.1017/S0950268821002375)
Supplement: Supplementary file 1 [file hygsup.zip › S0950268821002375sup001.docx]

| **Table 4.** The smoothing weight of exponential smoothing model. | | | | |
| --- | --- | --- | --- | --- |
| Parameter | Estimated value | Standard error | *t* statistic | *P* value |
| India |  |  |  |  |
| Horizontal smoothing weight | 0.829 | 0.056 | 14.900 | < 0.001 |
| Trend smoothing weight | 0.128 | 0.027 | 4.790 | < 0.001 |
| Seasonal smoothing weight | 0.761 | 0.269 | 2.830 | 0.005 |
| the US |  |  |  |  |
| Horizontal smoothing weight | 0.353 | 0.031 | 11.330 | < 0.001 |
| Trend smoothing weight | 0.060 | 0.022 | 2.790 | 0.006 |
| Seasonal smoothing weight | 0.138 | 0.038 | 3.670 | < 0.001 |
