# Supplementary material for: Comparison of ARIMA, ES, GRNN and ARIMA–GRNN hybrid models to forecast the second wave of COVID-19 in India and the United States
Source: Epidemiol Infect. 2021 Nov 2;149:e240. doi: 10.1017/S0950268821002375 (PMC8632421; doi:10.1017/S0950268821002375)
Supplement: Supplementary file 1 [file hygsup.zip › S0950268821002375sup003.docx]

| **[Table](https://www.ncbi.nlm.nih.gov/pmc/articles/PMC4892637/table/pone.0156768.t001/) 1.** The ADF test of the transformed COVID-19 new cases series. | | | | |
| --- | --- | --- | --- | --- |
| Region | ADF test | | *t* statistic | *P* value |
| India | Augmented Dickey-Fuller test statistic | | -19.753 | < 0.001 |
| the US | Augmented Dickey-Fuller test statistic | | -30.964 | < 0.001 |
|  | Test critical values | 1% level statistic | -3.445 |  |
|  |  | 5% level statistic | -2.873 |  |
|  |  | 10% level statistic | -2.570 |  |
