# Supplementary material for: Comparison of ARIMA, ES, GRNN and ARIMA–GRNN hybrid models to forecast the second wave of COVID-19 in India and the United States
Source: Epidemiol Infect. 2021 Nov 2;149:e240. doi: 10.1017/S0950268821002375 (PMC8632421; doi:10.1017/S0950268821002375)
Supplement: Supplementary file 1 [file hygsup.zip › S0950268821002375sup004.docx]

| **Table 2.** The *AIC*, *SBC*, and *R^2^* of the three appropriate ARIMA models in India and the US. | | | |
| --- | --- | --- | --- |
| ARIMA model | *AIC* | *SBC* | *R^2^* |
| India |  |  |  |
| (3, 1, 3) (3, 1, 3) _14_ | 8569.512 | 8622.158 | 0.996 |
| (3, 1, 3) (1, 1, 1) _14_ | 8572.657 | 8609.104 | 0.995 |
| (3, 1, 1) (3, 1, 1) _14_ | 8572.757 | 8617.305 | 0.996 |
| the US |  |  |  |
| (3, 1, 3) (3, 1, 2) _14_ | 9372.966 | 9421.564 | 0.946 |
| (2, 1, 3) (0, 1, 1) _14_ | 9375.214 | 9403.563 | 0.947 |
| (2, 1, 1) (2, 1, 1) _14_ | 9375.798 | 9412.246 | 0.947 |
| *AIC:* Akaike information criterion; *SBC:* schwarz bayesian criterion. | | | |
