# Supplementary figures and images for: Comparison of ARIMA, ES, GRNN and ARIMA–GRNN hybrid models to forecast the second wave of COVID-19 in India and the United States
Source: Epidemiol Infect. 2021 Nov 2;149:e240. doi: 10.1017/S0950268821002375 (PMC8632421; doi:10.1017/S0950268821002375)

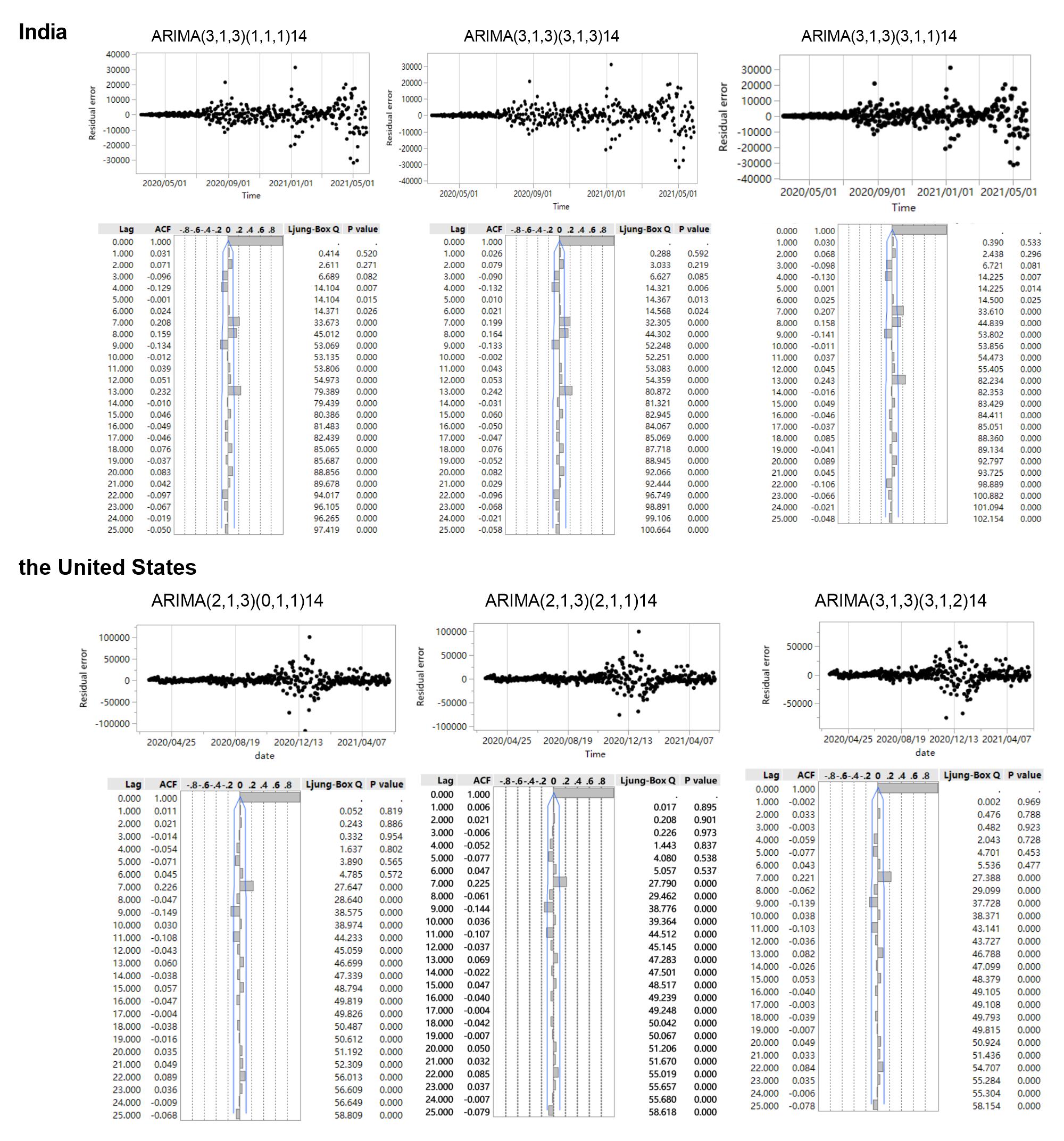

Supplement: Supplementary file 1 [file hygsup.zip › S0950268821002375sup002.jpg]
